# Clinical and Therapeutic Characteristics of Stroke and Their Implications on Outcomes: A Multi-center Study in Panama

**DOI:** 10.7759/cureus.78438

**Published:** 2025-02-03

**Authors:** Erick Villarreal, Harry Wolfschoon, Abdullah Salehji, Kayra Patiño, Denise Cardoze, Verónica Billingslea, Esteban González, Astrid Batista

**Affiliations:** 1 Faculty of Medicine, Universidad de Panamá, Panama, PAN; 2 Department of Hematology, Ciudad de la Salud, Caja del Seguro Social, Panama, PAN; 3 Department of Geriatrics, Complejo Hospitalario Metropolitano Dr. Arnulfo Arias Madrid, Caja del Seguro Social, Panama, PAN; 4 Department of Nephrology, Ciudad de la Salud, Caja del Seguro Social, Panama, PAN; 5 Department of Neurology, Ciudad de la Salud, Caja del Seguro Social, Panama, PAN; 6 Department of Internal Medicine, Hospital Gustavo Nelson Collado, Caja del Seguro Social, Chitré, PAN

**Keywords:** hemorrhagic stroke, inpatient mortality rate, intravenous thrombolysis, ischemic stroke, length of stay, outcomes, risk factors, stroke

## Abstract

Introduction

There are no previous studies that assess the time between stroke symptom onset and hospital arrival in Panama. With this study, we aim to describe the epidemiological, clinical, and therapeutic characteristics of the patients to determine whether our data on stroke care and outcomes align with those from other Latin American countries and nations with more advanced healthcare systems.

Methods

This prospective study included 214 stroke patients admitted to three healthcare centers in Panama between January and December 2023. Demographic, clinical, and therapeutic data were collected using a standardized instrument. Univariate analysis was then conducted in order to describe patient characteristics and their distribution across outcomes, which included discharge, critical care unit transfer, or death.

Results

Out of the 214 patients included in the study, 124 (57%) were men, and 88 (42%) were women. The main risk factors were arterial hypertension, type 2 diabetes, and previous history of stroke. The overall median time from symptom onset to arrival was 10 hours, and the median National Institutes of Health Stroke Scale (NIHSS) score on arrival was 5. The mean hospital stay was 10.4 days, and overall mortality was 38 (17.7%). Only five (2%) patients were able to receive intravenous thrombolysis.

Discussion

This study is the first to report the time from stroke symptom onset to hospital arrival in Panama, with a median recorded time of 10 hours. Despite national improvements in the length of stay and overall mortality, the negative results displayed by our study may be explained by the significant strain public hospitals face due to the high demand for their services.

Conclusion

The measured time in hours from symptom onset to arrival at a healthcare center across three centers in Panama was 10 hours, and thrombolysis was only recorded in five (2%) cases. Results reveal notable regional disparities and prompt further research on effective improvement strategies.

## Introduction

The World Health Organization (WHO) defines a cerebrovascular event (CVE) as a clinical syndrome of rapid development due to a focal disturbance of brain function of vascular origin and lasting more than 24 hours [1]. Generally, cerebrovascular disease is the second leading cause of mortality worldwide, accounting for 5.5 million deaths per year. Since 2010, there has been a global increase in reports of cerebrovascular events (CVE), with a prevalence and incidence of 322 and 156 per 100,000 people, respectively, reported in 2016. The frequency of CVE varies depending on region, race, sex, and age [2]. In Panama, according to data from the National Institute of Statistics and Census (INEC), cerebrovascular disease was the fifth leading cause of death nationwide [3]. Comparatively, this etiology is the third leading cause of death in Colombia and the fifth in Ecuador [4,5]. It represents the greatest cause of mortality and discapacity worldwide. In Panama, according to data from the World Health Organization, cardiovascular disease represents 23% of all mortality causes in men and 29% in women between ages 30 and 69 in the year 2014 [6].

There are multiple risk factors associated with cerebrovascular events, which could be non-modifiable such as age, sex, and genetic factors. But there are also modifiable risk factors such as arterial hypertension, diabetes mellitus, sedentary lifestyle, obesity, smoking, alcoholism, dyslipidemia, and cardiac disease [7-10]. The management of cerebrovascular events begins with the recognition of suggestive signs and continues with the activation of stroke protocols that approach the patient from the prehospital phase to ensure prompt and appropriate treatment [11,12].

No prior studies have evaluated the critical time between symptom onset and hospital arrival in Panama; furthermore, the last study that evaluated the quality of stroke care dates back a decade. This study aims to address these gaps by examining the epidemiological, clinical, and therapeutic characteristics of stroke patients. The description of these factors is essential in the development of national stroke protocols and the improvement of stroke care.

## Materials and methods

Study design

This prospective, observational, and descriptive multi-center study was conducted at three hospitals in Panama between January and December 2023. The main study site is the Dr. Arnulfo Arias Madrid Hospital Complex (CHDrAAM), a public tertiary care hospital located in the Panama capital. The hospital lacks a dedicated stroke unit, and care on admission is provided by the Internal Medicine Department. The remaining sites include the Gustavo Nelson Collado Hospital (HGNC), a regional hospital located in the Herrera province, and the Dr. Rafael Estévez Regional Hospital (HRRE), a regional hospital located in the Coclé province [13]. Both of these centers, located in the countryside, provide 24-hour emergency services to their respective areas.

Data on stroke care were prospectively collected via the interrogation and examination of patients; if needed, family members were also consulted. Demographic variables recorded were the following: age, sex, education level, and occupation. Clinical variables of interest were the following: time since symptom onset, signs and symptoms of stroke at arrival, vital signs, National Institutes of Health Stroke Scale (NIHSS), and laboratory parameters on arrival. Clinical manifestations assessed were the following: paresis or plegia; the term under which monoparesis, hemiparesis, paraparesis, and quadraparesis or plegia were all included; dysarthria; seizures; gait disorder; sensory and motor aphasia; headache; neurological deterioration; visual disorder; and syncope.

Suspected or known cardiometabolic and behavioral risk factors identified and recorded were the following: hypertension, obesity, type 2 diabetes, sedentary lifestyle (defined as less than an hour of moderate-intensity exercise per week), previous stroke, significant alcohol consumption (which was defined as more than four drinks per day or more than 14 drinks per week for men and more than three drinks per day or more than seven drinks per week for women as per the American Heart Association/American Stroke Association [14]), smoking, and atrial fibrillation.

Patients were followed throughout their hospital stay until three outcomes of interest were reported: discharge, intensive care unit (ICU) transfer, or death. In order to analyze the distribution of patient characteristics throughout outcomes, patients were divided into four subgroups according to outcomes: "survived," "did not survive," "ICU transfer," and "NA." Since follow-up was lost when they transferred to the ICU unit, these patients were included in a separate category and are displayed as such in tables. Furthermore, patients who did not have outcomes clearly described were included within the not accounted (NA) category. These details should be taken into consideration when interpreting results. Aspirin, statin, thromboprophylaxis, and intravenous thrombolysis use was also recorded in order to characterize treatment.

Patient population

Patients over the age of 18 with a suspected or confirmed stroke diagnosis admitted to the internal medicine and geriatric units of specified centers between January and December 2023 were included in the study. Data were recorded using a standardized collection instrument, and cases in which more than half of the required data points were missing or incomplete were excluded from the study. Due to the limitations of data collection methods, the characteristics of critical patients on arrival could not be adequately assessed, and standardized instruments could not be filled in many of these cases. Furthermore, data on patients who did not meet inclusion criteria are detailed in the Results section to be taken into consideration during interpretation.

Statistical analysis

All data were compiled and collected into a master chart accessible only to researchers that were then introduced into R software version 4.4.2 (R Foundation for Statistical Computing, Vienna, Austria) where data analysis was conducted. Univariate analysis was conducted for the demographic, clinical, and therapeutic characteristics and is as follows: continuous variables were summarized as mean ± standard deviation or median with first and third interquartile values (Q1 and Q3) according to data distribution. Categorical variables were presented as the total number of observations and their respective frequencies. Variables were assessed according to outcomes of interest and stroke type. Statistical significance was determined using Pearson's chi-squared test and Fisher's exact test when assessing categorical variables, Kruskal-Wallis rank sum test was used to evaluate multiple continuous variables. Wilcoxon rank sum test assessed multiple independent groups. P-values were considered significant when less than 0.05 and very significant when less than 0.001.

Ethical consent

A research protocol was developed where study design, data collection, and analysis methods were described in detail and was submitted for review to the Institutional Review Board of Dr. Arnulfo Arias Madrid Hospital Complex. No personal patient details were recorded, and data were handled with strict adherence to ethical guidelines in order to ensure confidentiality, and a waiver of consent was solicited. Approval was received from the National Research Bioethics Committee, and the following is the approval number: DENADOI-SIBI091-2022.

## Results

Data were prospectively collected from 214 patients who met the inclusion criteria and were admitted to the internal medicine and geriatric units with a cerebrovascular event diagnosis from three separate medical centers across the country. Five patients did not meet the inclusion criteria due to data collection methods: Three (60%) were from the Dr. Rafael Estévez Regional Hospital (HRRE), one (20%) patient was from the HGNC, and one (20%) was from the CHDrAAM. Four (80%) were stable on arrival, and the outcome was not specified. One (20%) was unstable on arrival and did not survive. Demographic data were recorded, and the following were the results (Table 1): 168 (78.5%) patients were from Dr. Arnulfo Arias Madrid Hospital Complex (CHDrAAM) located in the capital; 29 (13.5%) were from Gustavo Nelson Collado Hospital (HGNC), a hospital located in the countryside province of Herrera; and the remaining 17 (7.9%) were from Dr. Rafael Estévez Regional Hospital (HRRE) of the Chiriquí province, the second biggest province of Panama. Out of the 214 patients included in the study, 124 (57%) were men, and 88 (42%) were women; two (1%) were not accounted for. The cerebrovascular event was then characterized as ischemic, 159 (74%); hemorrhagic, 35 (16%); and transient ischemic attack, 14 (6.5%); six (2.8%) were not accounted for and were thus excluded from the stroke subtype analysis. The mean age of patients included in the study was 69 ± 14. One hundred twelve (52%) of the patients had only reached a high school level of education, 64 (29%) had only reached primary school, and 23 (11%) had gone into college. One hundred thirty-four (63%) were from Panama (capital), 27 (13%) were from Herrera, and 26 (12%) were from Panama Oeste province. This speaks only of the primary sites of data collection. Sixty-five (32%) of the patients registered were already retired, 43 (21%) were pensioned/disabled, 43 (21%) were housewives, and 37 (18%) were active workers.

**Table 1:** Demographic characteristics of patients included ^1^Mean ± SD; n (%) CHDrAAM, Dr. Arnulfo Arias Madrid Hospital Complex; HGNC, Gustavo Nelson Collado Hospital; HRRE, Dr. Rafael Estévez Regional Hospital; SD, standard deviation

Characteristic	N = 214^1^
Mean age (years)	69 ± 14
Sex	
Female	88 (42%)
Male	124 (57%)
Missing	2 (1%)
Hospital	
CHDrAAM	168 (79%)
HGNC	29 (14%)
HRRE	17 (7.9%)
Education level	
Primary school	64 (29%)
High school	112 (52%)
College	23 (11%)
Missing	15 (7%)
Occupation	
Housewife	43 (20%)
Unemployed	15 (7%)
Independent	6 (2%)
Retired	65 (30%)
Pensioned	43 (20%)
Active worker	31 (14%)
Missing	11 (5.1%)

**Figure 1:**
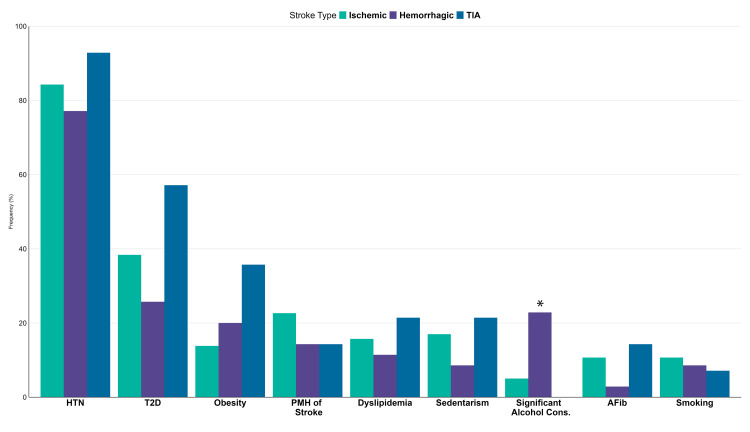
Distribution of risk factors by stroke type *Significant at p-value of <0.05. P-values are based on Pearson's chi-squared test or Fisher's exact test TIA, transient ischemic attack; HTN, hypertension; T2D, type 2 diabetes; AFib, atrial fibrillation; Cons, consumption; PMH, past medical history

As illustrated in Figure 1, the main risk factor across all subtypes of cerebrovascular event was arterial hypertension with 174 (84%) having a previous diagnosis, followed by type 2 diabetes with 78 (38%) patients suffering from it and a history of a previous stroke in 43 (21%) patients; sedentary lifestyle (33, 16%) and dyslipidemia (32, 15%) were also recorded risk factors. Significant alcohol consumption was more frequently noted in patients with hemorrhagic stroke with eight (23%) of the patients with the said subtype reporting it. This finding was significant (p = 0.004).

**Figure 2:**
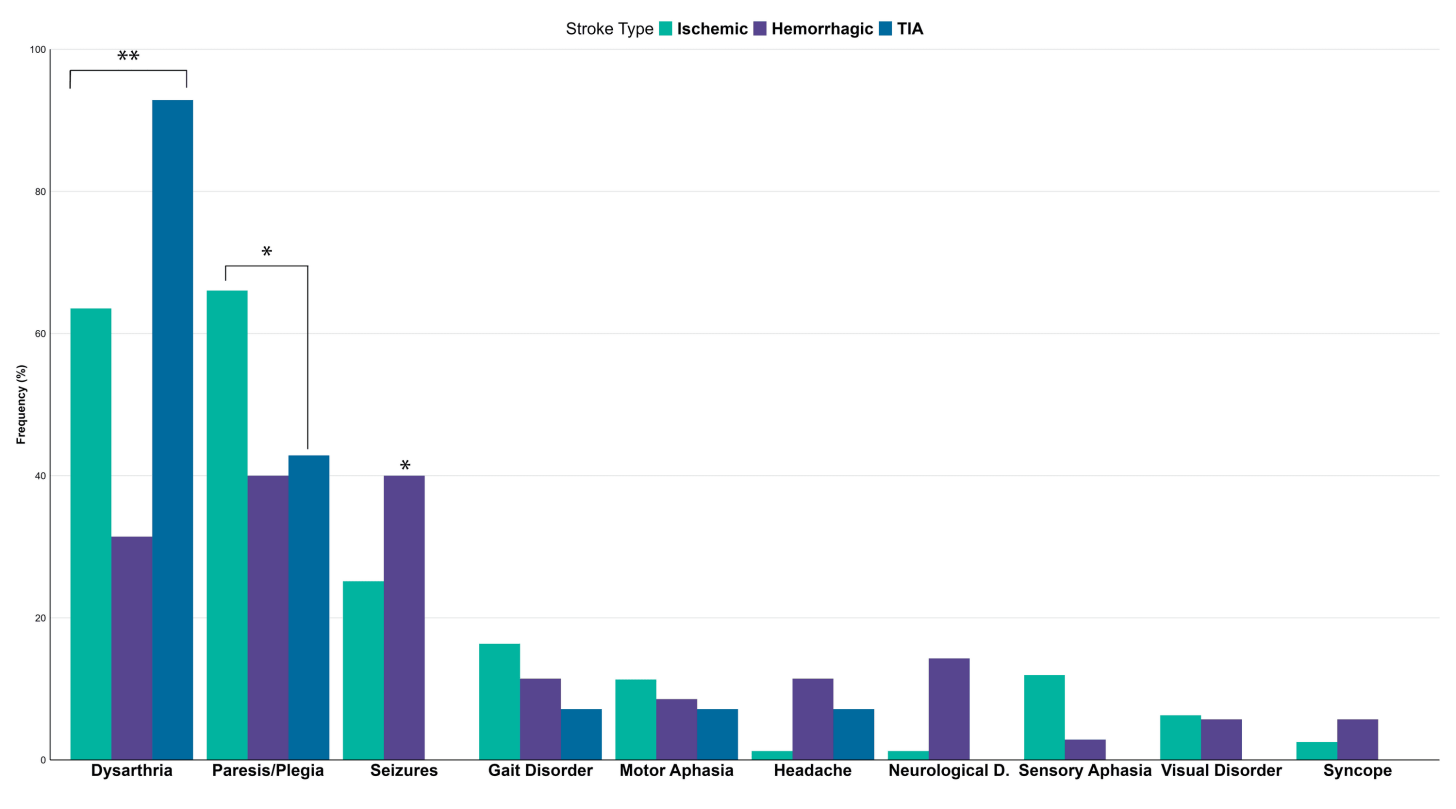
Distribution of clinical signs by stroke type *Significant at a p-value of <0.05 **Significant at a p-value of <0.001. P-values are based on Pearson's chi-squared test or Fisher's exact test TIA, transient ischemic attack; neurological D, neurological deterioration

The most common clinical manifestations reported by patients were paresis or plegia (125, 60%) and dysarthria (125, 60%), followed by seizures in 54 (26%) patients and abnormal gait in 31 (15%). Sensory and motor aphasia were also recorded presentations of patients. Dysarthria was more frequent in transient ischemic attack (p < 0.001), whereas paresis/plegia was more common in ischemic stroke (p = 0.007). Neurological deficit and headache were more frequent in hemorrhagic stroke (p = 0.003 and p = 0.0012, respectively). Further characterization can be seen in Figure 2.

**Figure 3:**
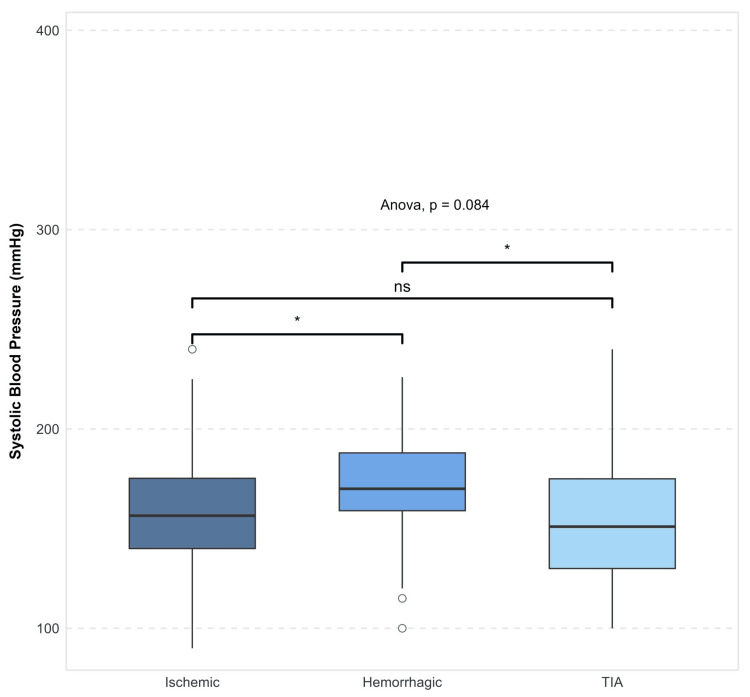
Systolic blood pressure distribution by stroke type *Significant at a p-value of <0.05. Based on ANOVA and pairwise comparisons TIA, transient ischemic attack; ns, not significant

The mean systolic blood pressure value on admission was 162 ± 30 mmHg, and the mean diastolic blood pressure was 90 ± 16 mmHg across all subtypes of cerebrovascular disease. Systolic blood pressure values were significantly increased (Figure 3) in hemorrhagic stroke with a mean of 171 ± 30 mmHg and a p-value of 0.0084.

The median Glasgow Coma Scale (GCS) score on presentation was 14 across all subtypes. It was significantly (p < 0.001) decreased in hemorrhagic stroke with a median of 12 (8 and 15). Paraclinic parameters were also recorded, and the following were the results: the mean hemoglobin (Hb) on admission tests was 13.02 ± 6.13, the mean hemoglobin A1C (HbA1C) was 6.73 ± 2.15%, and it was significantly increased in ischemic stroke with a mean of 6.87 ± 2.25% and a p-value of 0.038. The mean total cholesterol on admission was 187 ± 51 g/dL, and the mean low-density lipoprotein (LDL) was 115 ± 47 g/dL.

Our study is the first to report the time between symptom onset and arrival at a healthcare center in Panama (Table 2). The median time in hours from onset to arrival across all three tertiary care centers was 10 hours, with times being significantly higher in the ischemic subtype (median = 12 hours, p = 0.019) and the Dr. Arnulfo Arias Madrid Hospital Complex (median = 12, p = 0.034). The median (p25 and p75) NIHSS score was 5 (3 and 10) on arrival and 5 (2 and 9) on discharge. Patients were hospitalized for a median (Q1 and Q3) time in days of seven (four and 13) with times being significantly shorter in countryside hospitals (p = 0.009). Treatment characterization revealed that 124 (59%) patients received aspirin during admission, 135 (64%) received statins, 108 (51%) received thromboprophylaxis, and only five (2%) patients received thrombolysis; the other 206 (98%) did not. The most frequent localization in imaging studies was the basal ganglia, followed by the parietal, temporal, and frontal lobes.

**Table 2:** Characteristics of patients on admission according to stroke type ^1^Median (Q1 and Q3); n (%) ^2^Based on Pearson's chi-squared test or Fisher's exact test for categorical variables and Kruskal-Wallis rank sum test for continuous variables *Significant at a p-value of <0.05 **Significant at a p-value of <0.001 NIHSS, National Institutes of Health Stroke Scale; GCS, Glasgow Coma Scale; TIA, transient ischemic attack

Characteristic	Overall, N = 214^1^	Stroke subtype			P-value^2^
		Ischemic, N = 159^1^	Hemorrhagic, N = 35^1^	TIA, N = 14^1^	
Time since symptom onset (hours)	10 (six and 24)	12 (seven and 24)	Eight (six and 18)	Seven (five and 12)	0.019*
NIHSS on admission	5 (3 and 10)	5 (3 and 10)	12 (8 and 15)	1 (0 and 2)	0.006*
GCS on admission (median)	14.00 (11.00 and 15.00)	14.00 (11.00 and 15.00)	12.00 (8.00 and 15.00)	15.00 (15.00 and 15.00)	<0.001**
Median hospital stay (days)	Seven (four and 13)	Seven (four and 14)	Eight (four and 13)	Five (two and 11)	0.2

Outcomes were also recorded with overall 161 (75%) patients being discharged home, 38 (17.7%) patients dying during their stay, nine (4.7%) being transferred to the ICU, and six (2.8%) being not accounted for. The analysis of complications revealed that the most frequent was hospital-acquired infections with 53 (25%) suffering from them, followed by seizures (23, 11%) and the need for invasive mechanical ventilation (14, 6.7%).

Further subgroup analysis on the 38 (17.7%) patients who died in comparison to the 161 (75%) who had been discharged or transferred to the ICU (nine, 4.7%) was conducted (Table 3). As previously stated, follow-up was lost when they transferred, and ICU patients were thus included in a separate category. The results revealed that the mean age of surviving patients was 68 ± 13 years, whereas patients who did not survive had a mean age of 77 ± 11 years. Patients transferred to the ICU had a mean age of 62 ± 14 years, and patients not accounted for had a mean of 72 ± 10 years. The difference between groups was significant (p = 0.001). The NIHSS on admission was significantly higher in patients who did not survive with a median score of 12 (7 and 21) or had to be transferred to the intensive care unit (9 {8 and 10}), in comparison to a median of 5 (3 and 9) in patients who survived. The Glasgow Coma Scale score on arrival was also significantly different between the subgroups, with it being a median of 8.5 (6 and 11) in the patients who died and 15 (13 and 15) in the patients who survived. The group who survived had significantly higher rates of use of aspirin, statin, thromboprophylaxis, and thrombolysis than the group who did not. The subgroup who did not survive had significantly higher rates of infections, seizures, need for mechanical ventilation, and hemorrhagic transformation (p-values of <0.001, <0.001, <0.001, and 0.001, respectively).

**Table 3:** Distribution of clinical and therapeutic parameters across outcomes ^1^Mean ± SD; n (%); median (Q1 and Q3) ^2^Based on Pearson's chi-squared test or Fisher's exact test for categorical variables and Kruskal-Wallis rank sum test for continuous variables *Significant at a p-value of <0.05 **Significant at a p-value of <0.001 TIA, transient ischemic attack; GCS, Glasgow Coma Scale; IMV, invasive mechanical ventilation; NA, not accounted; NIHSS, National Institutes of Health Stroke Scale; ICU, intensive care unit; SD, standard deviation

Characteristic	Did not survive, N = 38^1^	Survived, N = 161^1^	ICU, N = 9^1^	NA, N = 6^1^	P-value^2^
Age (years)	77 ± 11	68 ± 13	62 ± 14	72 ± 10	0.001**
Time since onset (hours)	18 ± 17	16 ± 18	13 ± 15	11 ± 6	0.6
Stroke type					0.12
Ischemic	27 (73%)	121 (78%)	6 (67%)	5 (83%)	
Hemorrhagic	10 (27%)	21 (13%)	3 (33%)	1 (17%)	
TIA	0 (0%)	14 (9.0%)	0 (0%)	0 (0%)	
NIHSS on admission	12 (7 and 21)	5 (3 and 9)	16 (14 and 26)	4 (4 and 8)	<0.001**
GCS	8.50 (6.00 and 11.00)	15.00 (13.00 and 15.00)	9.00 (8.00 and 10.00)	14.50 (13.00 and 15.00)	<0.001**
Complications					
Infections	18 (47%)	27 (17%)	3 (33%)	5 (83%)	<0.001**
Seizure crisis	10 (26%)	9 (5.6%)	2 (22%)	2 (33%)	<0.001**
IMV need	6 (16%)	1 (0.6%)	7 (78%)	0 (0%)	<0.001**
Hemorrhagic transformation	5 (13%)	1 (0.6%)	0 (0%)	1 (17%)	0.001**
Thrombolysis					0.034*
No	38 (100%)	155 (98%)	7 (78%)	6 (100%)	
Yes	0 (0%)	3 (1.9%)	2 (22%)	0 (0%)	

## Discussion

We studied the demographic, clinical, and therapeutic characteristics and factors associated with worse outcomes of 214 patients of multiple tertiary care centers in Panama. The mean age of patients included in our study was similar to older national reports [15] and reports from neighboring countries [16]. Sex distribution did seem to change over the years, with a higher proportion of men suffering from cerebrovascular events [15]. It has been previously suggested that education years may act as a protective factor against stroke [17]. Notably, 112 (52%) of the patients included in our study who suffered cerebrovascular disease had only reached a high school level of education.

The most reported risk factors in our report were as follows: hypertension, diabetes mellitus, and previous stroke. These results are similar to those reported in North America, although dyslipidemia plays a bigger role [18]. Significant alcohol consumption has been previously linked to higher rates of hemorrhagic stroke, a finding supported by the higher count of alcohol consumption noted in patients with hemorrhagic stroke [19]. Falls were not accounted for in these patients and may play a role in the results. Further studies should be performed in this area.

The mean hospital stay of 10.4 days and the overall mortality rate of 38 (17.7%) had a significant decrease from older reports [15]. Although the rate of complications increased. This may be explained by the fact that despite being referral centers, none of the included hospitals have a dedicated stroke unit and patient care on admission is provided by the Internal Medicine and Geriatric Department. It has been previously stated that independent stroke units reduce the rate of complications [20]. Findings further establish the need for the development of dedicated stroke units in our country. These results also differ from those in developed countries where mortality falls as low as 5% and the length of stay ranges from eight to 16 days [21].

Older ages, higher NIHSS scores, and lower Glasgow Coma Scale scores on admission were reported on patients with worse outcomes. Older ages have been previously reported to be associated with higher in-hospital mortality [22]. The mortality of patients with hemorrhagic stroke was higher where 10 (28.57%) out of the 35 patients recorded did not survive. In contrast, 27 (16.98%) out of the 159 patients with ischemic stroke died. Hemorrhagic stroke patients also had higher NIHSS values on arrival in comparison to ischemic stroke. These results correlate to previous studies [23]. Blood pressure was not significantly different on admission among patients who did or did not survive. Time from symptom onset to hospital arrival was higher in those who had a worse prognosis, although this result was not significant.

As previously mentioned, the most significant result concluded by our study was the time it takes from symptom onset to arrival at the center. The median time from onset to arrival was 10 hours. The results stated in our study may be explained by the significant challenges public hospitals face due to the high demand for their services, with the total number of patient visits to the Emergency Department of the CHDrAAM reaching over 44,000, reflecting the negative effects of current health infrastructure and the pressing need for improvement [24]. They may also be explained by a lack of public awareness of stroke, which is yet to be assessed in the Panamanian population and may be an area of future research and improvement. The decreased public awareness of stroke symptoms has been previously linked to missed therapeutic windows [25].

These results are lower than those reported by studies from Peru, which cite being unaccompanied at the time of onset as the most frequent cause of hospital arrival delay. In Chile, the median time to arrival was eight hours and 11 minutes [26]. These results differ from studies published in Europe, where in 2017 the median prehospital delay was 201 minutes [27]. Factors associated with increased times are a lack of public awareness of the symptoms of stroke and delays in emergency medical services (EMS) arrival [28]. Accordingly, only five (2%) patients were able to receive intravenous thrombolysis for acute ischemic stroke. This reflects significant regional disparities influenced by health infrastructure, economic status, and cultural factors [29]. In comparison to other countries, Panama's thrombolysis rate is notably low. For example, high-income countries report higher utilization rates, with some studies reporting rates as high as 11.7% [30]. This disparity is even more pronounced when comparing it to countries from Central and North Asia [31].

Limitations

Our study faced significant limitations, the most important being the challenges in data collection, which included a lack of electronic medical records and a widespread registry of patient care data. This is reflected in the high number of missing values throughout the analysis, limiting the generalizability of results. Comorbidities were not accounted for in the analysis of outcomes and may play a role in the negative results observed. Further studies should take this into account. Socio-economic level was not taken into account in the present study and could also play a role. Data on hospital arrival times were also based on patient narratives, many of whom were unsure about their condition. No research was performed on the cause of delays in care; these may be as follows: slow emergency service response times, the lack of awareness, or logistical issues in included hospitals. Despite the shortcomings of our study, we hope that the data collected serve as a foundation for future research on stroke care, as this is the first of its kind to be developed on a national level.

## Conclusions

In this observational analysis across three centers in Panama, the average time from stroke onset to hospital arrival was 10 hours, with a low rate of thrombolysis in ischemic stroke patients. While mortality and the length of stay for stroke patients have decreased over time, the rate of complications has increased. These findings highlight significant regional disparities and underscore the urgent need for enhanced public awareness of stroke, improved healthcare infrastructure, and the development of national stroke protocols. Further research should be performed on the cause of hospital arrival delays and effective improvement strategies.
